# Supplementary material for: A multidisciplinary Prematurity Research Cohort Study
Source: PLoS One. 2022 Aug 25;17(8):e0272155. doi: 10.1371/journal.pone.0272155 (PMC9409532; doi:10.1371/journal.pone.0272155)
Supplement: S1 Table — (DOCX) [file pone.0272155.s001.docx]

**Supplemental Table 1: Sleep and lifestyle data surveys collected from participants**

| **Survey/Questionnaire** | **Number of questions** | **Example question** | **When implemented** |
| --- | --- | --- | --- |
| Perceived Stress Scale (15) | 10 | In the last month, how often have you been upset because of something that happened unexpectedly? | Once per trimester |
| Munich Chronotype Questionnaire (16) | 4 | I have a regular work schedule (this includes being a house wife or a house husband). Y/N | Once per trimester |
| Pittsburgh Sleep Quality Index (17) | 9 | During the past month, how often have you taken medicine to help you sleep (prescribed or “over the counter”)? | Once per trimester |
| Berlin Questionnaire (18) | 10 | Have you ever nodded off or fallen asleep while driving a vehicle? | Once per trimester |
| Women’s Health Initiative Insomnia Rating Scale (19) | 5 | In the past 4 weeks, did you wake up several times at night? | Once per trimester |
| Epworth Sleepiness Scale (20) | 8 | How sleepy are you while sitting and reading? | Once per trimester |
| International Restless Legs Syndrome Rating Scale (21) | 5 | Do you experience an urge to move or unpleasant sensations that begin or worsen during periods of rest or inactivity such as laying or sitting? | Once per trimester |
| Kaiser Physical Activity Survey (22) | 29 | How many minutes a day do you usually walk and/or bicycle to and from work, school or errands? | Once per trimester |
| Edinburgh Postnatal Depression Scale (23) | 10 | I have been able to laugh and see the funny side of things. (As much as always, not quite so much now, definitely not so much, not at all) | Once per trimester |
| Difficult Life Circumstances  (24) | 30 | Are you having regular arguments or conflicts with your present partner? | Second trimester |
| National Institutes of Health Diet History Questionnaire II (25) | 153 | Over the past 12 months, how often did you drink carrot juice? | Third trimester or within three months of delivery |
| Demographic and medical history questions | 11 | At what age did you have your first menstrual period? | First trimester |
| Lifestyle questions | 12 | Do you currently smoke cigarettes? | Once per trimester |
