# Supplementary material for: A multidisciplinary Prematurity Research Cohort Study
Source: PLoS One. 2022 Aug 25;17(8):e0272155. doi: 10.1371/journal.pone.0272155 (PMC9409532; doi:10.1371/journal.pone.0272155)
Supplement: S1 File — (DOC) [file pone.0272155.s002.doc]

**1000-Women Cohort Study**

**Demographic Questions**

Study ID__________ Date Completed: ___________________

| 1. **What is the highest level of education (school) that you have completed? (Check one)**  - Less than 12th grade - High school degree/GED - Some college/vocational school - College degree (4 years) - Graduate degree |
| --- |
| 1. **Do you currently have a paid job (full-time or part-time)? (check one)**  - Yes (Continue to question 2a) - No (Skip to question 3)   **2a. If yes, how many hours per week do you usually work at this job?**   - Less than 20 hours per week - 21- 30 hours per week - 31-40 hours per week - More than 40 hours per week   **2b. For how many years have you been working at this job?**   - Less than 1 year - Between 1 and 2 years - Between 2 and 3 years - Between 3 and 4 years - More than 4 years |
| 1. **What is your total annual household income (in US dollars) before taxes? (check one)**  - Below $15,000 - $15,000 - $24,999 - $25,000 - $49,999 - $50,000 - $74,999 - $75,000 - $99,999 - $100,000 - $124,999 - $125,000 - $149,999 - $150,000 - $199,999 - Greater than or equal to $200,000 - Government assistance such as WIC program, TANF, Food Stamps or other non-taxable income   **3a. How many people including you are supported by the income indicated above? (circle one)**  1 2 3 4 5 6 7 8 9 10 11 12 13 14 or more |
| **4. How many days do you experience between periods?** (from first day of period to start of next period)   - Less than 20 days - 21 to 27 days - 28 to 35 days - 36 days or more - Don’t know |
| **5. At what age did you have your first menstrual period?**   - Less than 10 years old - 11 to 14 years old - 15 years or older - Don’t know |
| **6. When you are not on any birth control, are your periods regular?**   - Yes (Continue to question 6a) - No (Skip to question 7) - Don’t know (Skip to question 7)   **6a. How old were you when your menstrual periods became regular?**   - Less than 10 years old - 11 to 14 years old - 15 to 18 years old - 19 years or older - Don’t know |
| **7. How many days does your menstrual period usually last without the use of birth control**  **(bleeding, not spotting)?**   - 1 to 3 days - 4 days - 5 days - 6 days - 7 or more days - Don't know |
| **8. Have you used birth control in the past?**   - Yes (Continue to question 8a) - No (Skip to question 9)   **8a. If yes, what kind of birth control did you use?**  List all used: _________________________________________________________________  **8b. In total, how many years total did you take birth contraceptives?**  Total # years for pills:_________________ Total # years for other: ______________________ |
| **9. Have you ever tried for twelve months or more to become pregnant without success?**   - Yes (Continue to question 9a) - No, I tried and was successful (Skip to question 10) - No, I have never tried to get pregnant before (Skip to question 10) - Don’t know (Skip to question 10)   **9a. If yes, how old were you? (check all that apply)**   - Less than 20 - 21 to 25 years old - 26 to 30 years old - 31 to 35 years old - 36 years or older - Don’t know |
| **10. Approximately how much did you weigh when you were 17 years old?**   - Less than 100 pounds - 101 to 130 pounds - 131 to 160 pounds - 161 to 190 pounds - 191 or more pounds |
| **11. Have you ever been to a sleep clinic?**   - Yes - No |

**1000-Women Cohort Study**

**Lifestyle Questions**

| **12. Do you currently smoke cigarettes?**   - Yes (Continue to question 12a) - No (Skip to question 13)   **12a. If yes, on average, how many cigarettes do you smoke per day?**   - Less than 5 cigarettes per day - 6 to 10 cigarettes per day - 11 to 20 cigarettes per day - 21 or more cigarettes per day   **12b. How old were you when you started smoking?**   - Less than 16 (Skip to question 14) - 17 to 19 years old (Skip to question 14) - 20 to 25 years old (Skip to question 14) - More than 25 years old (Skip to question 14) |
| --- |
| **13. If you do not smoke now, was there ever a time in the past that you smoked on a regular basis?**   - Yes (Continue to question 13a) - No (Skip to question 14) |
| **13a. If yes, how long ago did you quit?**   - Less than 6 months ago - 7 to 12 months ago - 13 to 18 months ago - More than 18 months ago   **13b. How many years did you smoke?**   - Less than 1 year - 1 to 3 years - 4 to 6 years - 7 years or more |
| **13c. How many cigarettes did you smoke per day, on average?**   - Less than 5 cigarettes per day - 6 to 10 cigarettes per day - 11 to 20 cigarettes per day - 21 or more cigarettes per day   **13d. How old were you when you started smoking?**   - Less than 16 - 17 to 19 years old - 20 to 25 years old - More than 25 years old |
| **14. Do you currently live with anyone who regularly smokes around you?**   - Yes - No |
| **15. Do you currently smoke e-cigarettes?** |
| - Yes (Continue to question 15a) - No (Skip to question 16)   **15a. If yes, about how many e-cigarettes do you smoke per day?**   - Less than 5 e-cigarettes per day - 6 to 10 e-cigarettes per day - 11 to 20 e-cigarettes per day - 21 or more e-cigarettes per day   **15b. If yes, how old you were when you started smoking e-cigarettes?**   - Less than 16 (Skip to question 17) - 17 to 19 years old (Skip to question 17) - 20 to 25 years old (Skip to question 17) - More than 25 years old (Skip to question 17) |
| **16. If you do not smoke e-cigarettes now, was there ever a time in the past that you smoked them**  **on a regular basis?**   - Yes (Continue to question 16a) - No (Skip to question 17)   **16a. If yes, how long ago did you quit?**   - Less than 6 months ago - 7 to 12 months ago - 13 to 18 months ago - More than 18 months ago   **16b. How many years did you smoke e-cigarettes?**   - Less than 1 year - 1 to 3 years - 4 to 6 years - 7 years or more     **16c. How many e-cigarettes did you smoke per day, on average?**   - Less than 5 cigarettes per day - 6 to 10 cigarettes per day - 11 to 20 cigarettes per day - 21 or more cigarettes per day   **16d. How old were you when you started smoking e-cigarettes?**   - Less than 16 - 17 to 19 years old - 20 to 25 years old - More than 25 years old |
| **17. Have you ever lived with anyone who smoked e-cigarettes around you regularly in the past?**   - Yes - No |
| **18. Do you currently use recreational or illegal drugs?**   - Yes (Complete table below question 18a) - No (Skip to question 19) |
| **18a. If yes** please complete the table below:   | **Type** | **Used?** | **If yes, how often? (Check one)** | **How old when**  **first used** | | --- | --- | --- | --- | | Marijuana | - Yes - No | - Never - At least once a day - At least one time a week (but not every day) - At least monthly (but not every week) | ________ Years old | | Hashish, Hashish Oil | - Yes - No | - Never - At least once a day - At least one time a week (but not every day) - At least monthly (but not every week) | ________ Years old | | Cocaine | - Yes - No | - Never - At least once a day - At least one time a week (but not every day) - At least monthly (but not every week) | ________ Years old | | Crack, Rock, Ice | - Yes - No | - Never - At least once a day - At least one time a week (but not every day) - At least monthly (but not every week) | ________ Years old | | Barbiturates, Hypnotics, or “Downers” | - Yes - No | - Never - At least once a day - At least one time a week (but not every day) - At least monthly (but not every week) | ________ Years old | | Amphetamines, (Cross‐tops, Whites, Beannies, “Uppers”) | - Yes - No | - Never - At least once a day - At least one time a week (but not every day) - At least monthly (but not every week) | ________ Years old | | Methamphetamines (Speed, Crank) | - Yes - No | - Never - At least once a day - At least one time a week (but not every day) - At least monthly (but not every week) | ________ Years old | | LSD or other Hallucinogens | - Yes - No | - Never - At least once a day - At least one time a week (but not every day) - At least monthly (but not every week) | ________ Years old | | PCP (Angel Dust, Sherm) | - Yes - No | - Never - At least once a day - At least one time a week (but not every day) - At least monthly (but not every week) | ________ Years old | | Heroin or other Opiates | - Yes - No | - Never - At least once a day - At least one time a week (but not every day) - At least monthly (but not every week) | ________ Years old | | Steroids | - Yes - No | - Never - At least once a day - At least one time a week (but not every day) - At least monthly (but not every week) | ________ Years old | | Pharmaceutical drugs not prescribed for  you | - Yes - No | - Never - At least once a day - At least one time a week (but not every day) - At least monthly (but not every week) | ________ Years old | | Other not listed.  Specify: ________ | - Yes - No | - Never - At least once a day - At least one time a week (but not every day) - At least monthly (but not every week) | ________ Years old | |
| **19. Are you adopted?**   - Yes - No - Don’t know |
| **20. Did your mother go through natural menopause?**   - Yes (Continue to question 20a) - No (Skip to question 21) - Don’t know (Skip to question 21)   **20a. If yes, at what age did your mother go through natural menopause?**   - Less than 40 years old - 41 to 45 years old - 46 to 50 years old - 51 to 55 years old - More than 55 years old - Don’t know |
|  |
| **21. Are you a twin?**   - Yes (Continue to question 21a) - No (Skip to question 22)     **21a. If yes, what kind of twin are you?**   - Identical - Fraternal - Don’t know |
| **22. Were you born prematurely?**   - Yes (Continue to question 22a) - No (Skip to question 23) - Don’t know (Skip to question 23)   **22a. If you were born prematurely, how many weeks was your mother when she had you?**   - Less than 27 weeks - 28 to 32 weeks - 33 to 36 weeks - Don’t know |
| **23. What was your birthweight**? ___________ pounds ______________oz.   - Don’t Know |
