# Supplementary material for: A multidisciplinary Prematurity Research Cohort Study
Source: PLoS One. 2022 Aug 25;17(8):e0272155. doi: 10.1371/journal.pone.0272155 (PMC9409532; doi:10.1371/journal.pone.0272155)
Supplement: Attachment — Submitted filename: Response to Reviewers_final.docx [file pone.0272155.s003.docx]

**Response to Reviewers**

**We thank the reviewers for the thorough and positive review of the manuscript. We have responded to all the comments below.**

**Reviewer #1:** Congratulations on an enormous logistical undertaking.

***How was the cohort size determined?*** A convenience sample size of 1000 participants was chosen as a balance between an aggressive enrollment target given annual delivery volumes, the need to recruit and retain participants in multiple projects, and the varied outcomes assessed in each project. No *a priori* power analysis was conducted. We added this information to the Methods section (Lines 93–96).

***Why were patients conceived via IVF excluded?*** IVF patients were excluded from this cohort because of the *a priori* increased risk of preterm birth and pregnancy complications. Further, it was unknown at the time whether the IVF process modified the endocrine physiology of pregnancy, including unknown impacts of fresh and frozen cycles on hormonal states.

***The authors state that all patients were offered enrollment into projects 1 and 2. How were patients selected for project 3?*** Patients meeting the following inclusion/exclusion criteria were offered enrollment into Project 3: Pre-pregnancy body mass index < 40kgm^2^, willing and able to come to all MRI study appointments, no claustrophobia, no metal implants or non-removable body piercings, and no plans for scheduled cesarean delivery. We added this information to the manuscript (Lines 182-185).

**Reviewer #2: *This study presents the methodology in establishing a large cohort to investigate preterm birth. Overall this is a well written and presents a well-designed and elaborated methodology in recruiting patients to the cohort. Since this manuscript only presents the methodology and does not reveal any of the actual results of that were investigated. I question its interest to the readers. Numerous studies have been previously published on preterm birth, some were prospective randomized and some had large cohorts. I believe the ingenuity of this study lies on the three presented investigated topics and less on the recruitment process. My main concern is whether publishing a prelim manuscript as presented here is even relevant and wonder if all this should just be embedded in the manuscripts publishing the results.***

We argue that the longitudinal approach and recruitment success in a diverse population warrants a separate manuscript independent of the results. Furthermore, the three projects for which this cohort was formed differ substantially from one another. Thus, our plan is to describe the cohort and recruitment process in this index manuscript and then refer back to this manuscript in all future publications that contain results from the projects and future efforts. We argue this approach is more reader friendly than embedding all the information into future individual manuscripts. This approach has been modeled by several other large-scale and longitudinal cohort studies in pregnancy and women’s health. Examples include PMIDs 34610322, 21819423, and 27459450.
